# Alcohol hand sanitisers on mental health wards safety risk educational and QI poster

**DOI:** 10.1192/bjo.2021.556

**Published:** 2021-06-18

**Authors:** Peter McMurray

**Affiliations:** Bluestone Unit SHSCT

## Abstract

**Aims:**

To provide awareness of safety concerns around use of alcohol hand sanitiser on a mental health ward, and to consider ways of improving how learning for a serious adverse incident in one trust can better be communicated to other trusts

**Background:**

DD a male patient with history of paranoid schizophrenia alongside historic illicit drug use and current alcohol dependency admitted detained to Bluestone hospital following bizarre behaviour at a wake. Had been non-compliant with medication. Transferred to PICU due to going AWOL and returning under influence of alcohol.

2nd April overnight staff noted him to become over-sedated, presenting with slurred speech and appeared under influence of alcohol – transferred to A + E due to deteriorating GCS – was intubated, and transferred to ICU. Blood alcohol level was 373. Several empty bottles of hand sanitiser from dispensers on ward found in his room and he later disclosed he had accessed further alcohol hand sanitiser in sluice while washing clothes

SAI learning outcomes from one healthcare trust in Northern Ireland not currently routinely shared with other trusts

**Method:**

Literature review carried out to search for reports of similar incidents – 1 previous review article suggesting one death and 11 other major complications from consumption of alcohol hand sanitiser over 5 year period 2005-2009.

Quality improvement steps implemented to address this risk

Ward policy was reviewed to ensure patients no longer had unsupervised access to wash clothes

Liaised with Infection Control to assess the need for alcohol hand sanitiser to be available to patients given the ward is effectively a community setting

Intoxication policy reviewed and education sessions on this provided to all medical and nursing staff

Regional regular PICU staff update seminar launched for purpose of bringing PICU staff from across Northern Ireland together to share learning from SAIs and cases

**Result:**

Infection control agreed alcohol hand sanitiser dispensers could be removed from wards and kept only in locked nursing office with use of visitors.

Learning from this case shared with other trusts locally at newly launched regional PICU update seminar

No further incidents to date

**Conclusion:**

Patient access to alcohol hand sanitisers found to be a significant safety risk in PICU setting

Following implementation of quality improvement steps no further incidents of patients swallowing alcohol hand sanitiser

Improved awareness of risk of alcohol intoxication on ward with nursing staff escalating concerns to on-call doctor more frequently

